# Competition and specialization in an African forest carnivore community

**DOI:** 10.1002/ece3.5391

**Published:** 2019-08-27

**Authors:** David R. Mills, Emmanuel Do Linh San, Hugh Robinson, Sam Isoke, Rob Slotow, Luke Hunter

**Affiliations:** ^1^ School of Life Sciences University of Kwazulu‐Natal Durban South Africa; ^2^ Panthera New York New York USA; ^3^ Department of Zoology and Entomology University of Fort Hare Alice South Africa; ^4^ Wildlife Biology Program, W.A. Franke College of Forestry and Conservation University of Montana Missoula Montana USA; ^5^ Wildlife Conservation Society Kampala Uganda; ^6^ Department of Genetics, Evolution and Environment University College London London UK

**Keywords:** avoidance, interference, niche, occupancy, small carnivore, temporal

## Abstract

Globally, human activities have led to the impoverishment of species assemblages and the disruption of ecosystem function. Determining whether this poses a threat to future ecosystem stability necessitates a thorough understanding of mechanisms underpinning community assembly and niche selection. Here, we tested for niche segregation within an African small carnivore community in Kibale National Park, Uganda. We used occupancy modeling based on systematic camera trap surveys and fine‐scale habitat measures, to identify opposing preferences between closely related species (cats, genets, and mongooses). We modeled diel activity patterns using kernel density functions and calculated the overlap of activity periods between related species. We also used co‐occupancy modeling and activity overlap analyses to test whether African golden cats *Caracal aurata* influenced the smaller carnivores along the spatial and/or temporal axes. There was some evidence that related species segregated habitat and activity patterns. Specialization was particularly strong among forest species. The cats and genets partitioned habitat, while the mongooses partitioned both habitat and activity period. We found little evidence for interference competition between African golden cats and other small carnivores, although weak interference competition was suggested by lower detection probabilities of some species at stations where African golden cats were present. This suggests that community assembly and coexistence in this ecosystem are primarily driven by more complex processes. The studied carnivore community contains several forest specialists, which are typically more prone to localized extinction. Preserving the observed community assemblage will therefore require the maintenance of a large variety of habitats, with a particular focus on those required by the more specialized carnivores.

## Graphical abstract

In African forests where leopards have been extirpated, the remaining carnivore community is structured by habitat preference rather than interference competition. This means that the African golden cat, the largest remaining carnivore in these communities, will not suppress populations of smaller carnivores, as has been observed in other communities where mesopredator release causes dramatic restructuring throughout communities of carnivores and their prey. Protection of forest habitat will therefore preserve the remaining forest carnivore community assembly.

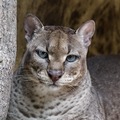

## 1 INTRODUCTION

The effective maintenance of biodiversity and ecosystem processes requires the preservation of diverse functional communities (Brose & Hillebrand, 2016; Connell & Ghedini, 2015; Supp & Ernest, 2014). To accomplish this, we need to understand the resource requirements of member species, and how these interact to form observed community assemblages (Diamond, 1975; Mittelbach & Schemske, 2015; Tilman, 2004). Furthermore, we need to understand the mechanisms underpinning individual species requirements relative to the rest of the community. While our understanding of community ecology has increased dramatically in recent decades (Mittelbach & Schemske, 2015; Pearson, Ortega, Eren, & Hierro, 2018; Trisos, Petchey, & Tobias, 2014), there remains a significant research gap in remote regions of the world, such as the Afrotropical forest biome.

Ecological communities are structured through multiple mechanisms including resource selection, interspecific competition, facilitation, and drift (Chesson, 2000; Connor & Simberloff, 1979; Diamond, 1975; Schoener, 1974). In theory, no two species should be able to occupy exactly the same niche (Giller, 1984; Pianka, 1981; Vanak et al., 2013). However, the situation is often far more complex. The theory of emergent neutrality suggests that species can occupy similar niches and that natural selection can produce groups of ecologically similar species (Sakavara, Tsirtsis, Roelke, Mancy, & Spatharis, 2018; Vergnon, Van Nes, & Scheffer, 2012). Even interspecific competition is not straightforward, since there is evidence that strong intraspecific competition in a dominant species can actually facilitate positive population growth in rare species (Chesson, 2000). Both natural and human‐derived perturbations to ecosystems can dramatically change how these processes influence community structure and function over time (Mouillot, Graham, Villéger, Mason, & Bellwood, 2013).

Carnivores (order Carnivora) are particularly sensitive to habitat change and are at risk of extinction and loss of functional diversity (Di Minin et al., 2016). Apex predators continue to disappear from ecosystems at an alarming rate, and small carnivores are exposed to increasing levels of disturbance (Di Minin et al., 2016; Ripple et al., 2014). Most research thus far has focused on large species (Ripple & Beschta, 2012; Wolfe et al., 2015). However, it is important to understand how different mechanisms influence the successful coexistence of entire carnivore communities, particularly poorly studied small species (Gompper, Lesmeister, Ray, Malcolm, & Kays, 2016). While small carnivore communities are now receiving more attention, African tropical forest communities remain poorly studied (Bahaa‐el‐din et al., 2013; Ray & Sunquist, 2001). These communities are diverse and relatively intact across much of the central African forest block, which is one of the highest priority areas for the expansion of the protected area network for mammalian carnivores (Kingdon & Hoffmann, 2013; Di Minin et al., 2016). However, extensive economic development is expected across the region, which will threaten the integrity of this community and its important functional role in the system (Edwards et al., 2014; Laurance et al., 2006). Understanding mechanisms underpinning the sensitivity and resilience of this community is essential to the design and management of any proposed conservation.

While we acknowledge current and past complexity in processes governing species coexistence, we focus on two basic processes as a starting point to understanding the relatively unstudied small carnivore communities in Africa's tropical forests. First, the theory of limiting similarity refers to an evolutionary process whereby closely related species that have similar resource requirements and competitive ability are expected to compete strongly, and completely segregate their niches through obligate resource specialization (Macarthur & Levins, 1967) (Table 1). While there is evidence for this theory, studies have demonstrated that observed community assemblages result from an interaction between these evolutionary processes and ecological processes, including aspects of fitness, niche availability, and trophic level (Fritschie, Cardinale, Alexandrou, & Oakley, 2014; Herben & Goldberg, 2014; Tilman, 2004).

**Table 1:** Processes and mechanisms driving community assembly

Process	Mechanism	Prediction	Observations from this study^a^	Conclusions
Limiting similarity	Closely related species strongly partition along one or more niche axes	Taxonomically related species will show opposing relationships in habitat preference and/or minimal overlap in core activity periods	Habitat: AGC–SVL, SG–RSG Temporal: MM–LGM	Related species strongly partition habitat or activity periods
Interference competition (occupancy)	Subordinate species completely avoid areas that a dominant species regularly uses	Occupancy probability of subordinate species will be lower in areas used by a dominant species	No effect	Small carnivores are not spatially displaced by African golden cats
Interference competition (detection)	Subordinate species are more cautious in areas used by a dominant species	Detection probability of subordinate species will be lower in areas used by a dominant species due to reduced trail use	Yes: AGC–AC; AGC–APC	Reactive temporary, ad hoc avoidance may occur between some species
Interference competition (temporal)	Subordinate species change activity periods to avoid times of peak activity of a dominant species	Core (50%) activity periods of subordinate species will not overlap with those of larger ones	Yes: AGC–AC; AGC–APC	Avoidance of peak activity periods only occurs in some species
Specialization (habitat)	Species evolve to exploit a specific subset of available biotic resources	Occupancy probability for a habitat specialist will be significantly influenced by one or more habitat variables	Forest: AGC, APC, SG, MM Nonforest: RSG No association (generalist): AC	Habitat specialization is important to varying degrees
Specialization (temporal)	Species establish activity patterns that maximize acquisition of preferred resources	Species will show strong diurnal, nocturnal, or crepuscular activity patterns	LGM, SM: diurnal; AGC, SVL: nocturnal and crepuscular; AC, APC, SG, RSG, MM: nocturnal	Nocturnal specialization is an important driver of community dynamics and interactions

a AC, African civet; AGC, African golden cat; APC, African palm civet; LGM, large gray mongoose; MM, marsh mongoose; RSG, rusty‐spotted genet; SG, servaline genet; SM, slender mongoose; SVL, serval (Figure 1).

Second, interference competition strongly influences species coexistence and realized niches within a community (Case & Gilpin, 1974; Rowles & O'Dowd, 2006; Zhang, Andersen, Dieckmann, & Brännström, 2015) (Table 1). The potential for lethal interactions with dominant species creates a landscape of fear among subordinate competing species and causes them to avoid certain habitats, times of the day, or food items (Gompper et al., 2016; Laundré et al., 2014; Rowles & O'Dowd, 2006). Subordinate generalist species may thereby become facultative specialists with a realized niche that is much narrower than their fundamental niche (Grassel & Rachlow, 2018).

Species co‐occurrence within a community is often partially achieved through resource specialization (Kassen, 2002). Competition for valuable resources causes some species to specialize, to a greater or lesser degree, in diet, habitat, and/or activity patterns (Alves, Diniz‐Filho, & Villalobos, 2017; Peers, Thornton, & Murray, 2012) (Table 1). Obligate specialization occurs through evolutionary processes, when a species evolves phenotypic traits that allow it to more efficiently exploit a narrow range of resources. These species tend to be better able to exploit resources within their specialization and are less prone to competitive exclusion, but they are more sensitive to perturbations because they cannot persist without their required resource (Burin, Kissling, Guimarães, Şekercioğlu, & Quental, 2016; Pardini, Bueno, Gardner, Prado, & Metzger, 2010). Conversely, generalist species have access to a greater variety of resources and are, therefore, more resilient to changes in the availability of any one particular resource (Gómez‐Rodríguez, Baselga, & Wiens, 2015). Facultative specialization occurs in generalist species when they are forced through competition to use a particular subset of the resources available to them within their fundamental niche (Burstahler, 2016; Grassel & Rachlow, 2018). These may not be preferred resources if high‐value resources are monopolized by a dominant competitor. When competition is removed, facultative specialists may expand their niche to include more resources or shift specialization to newly available high‐value resources (Alves et al., 2017). Where a species falls on the generalist–specialist gradient therefore influences its adaptability to environmental perturbations or disturbance. The presence of obligate specialists within an ecological community may reduce the resilience of its functional diversity (Clavel, Julliard, & Devictor, 2011).

We can use occupancy modeling to gain insight into mechanisms of niche specialization between closely related species (limiting similarity) or through ecological mechanisms (interference competition; Table 1). Single‐species occupancy modeling has become an important tool for identifying influential biotic and abiotic factors contributing to species presence and persistence in a given ecosystem (Braczkowski et al., 2016; MacKenzie et al., 2006; Steenweg et al., 2016). Two‐species occupancy modeling assists in the study of resource partitioning influenced by competition (MacKenzie, Bailey, & Nichols, 2004; MacKenzie et al., 2006; Richmond, Hines, & Beissinger, 2010). These techniques have provided important insights into habitat requirements, response to human activities, and interspecific community dynamics in a number of ecosystems (Farris, Kelly, Karpanty, & Ratelolahy, 2016; Gompper et al., 2016; Nagy‐Reis, Nichols, Chiarello, Ribeiro, & Setz, 2017; Schuette, Wagner, Wagner, & Creel, 2013).

Here, we test for evidence of interspecies competition along the temporal and spatial niche axes in a small carnivore community in a tropical forest in western Uganda. We compare niche separation between closely related species, and between African golden cats *Caracal aurata*, the dominant predator in the community and other small carnivores (cats, genets, and mongooses). Using camera trap surveys, we first establish community membership in our study area. The diets of the small carnivores included in this study overlap considerably, consisting mainly of rodents, with the possible inclusion of insects, fruit, and scavenged items (Kingdon & Hoffmann, 2013). Therefore, we focus here on determining whether each species is a generalist or specialist on both habitat and temporal axes, using single‐species occupancy models and modeling diel activity patterns. We expect the occupancy probability of habitat specialists to be significantly influenced by one or more habitat variables. We expect temporal specialists to demonstrate strict diurnal, crepuscular, or nocturnal, activity patterns, and generalists to be cathemeral. We then test for alternative mechanisms of community  assembly. Firstly, we test for niche partitioning between closely related species (limiting similarity), by determining whether occupancy probabilities of related species show strongly opposing relationships with habitat, and/or whether their diel activity patterns are strongly segregated. Secondly, we use two‐species occupancy models to indirectly assess interference competition, as indicated by spatial and temporal segregation between African golden cats and smaller carnivore species. If a strong effect is present, we expect occupancy probability of subordinate species to be lower when a dominant species is present. If the effect is weaker, we expect occupancy probability to remain unchanged, and detection probability to be lower when the dominant species is present. We test for temporal exclusion by comparing overlap in diel activity patterns. We expect subordinate species to avoid times when dominant species are most active. Finally, we discuss the potential impacts of perturbation and disturbance on community stability based on this mechanistic understanding of assembly rules.

## 2 METHODS

### 2.1 Study area

Kibale National Park (hereafter Kibale) is a 795 km^2^ mid‐altitude mosaic of tropical forest, regenerating forest, bush, grassland, and papyrus swamp, located in southwestern Uganda (Chapman & Lambert, 2000). Average minimum and maximum temperatures are 15.5°C and 23.7°C (Ryan, Chapman, & Rothman, 2013). Kibale experiences two rainy seasons, producing a mean of 1,701 mm of rainfall, and occurring roughly from March to May, and September to November (Chapman et al., 2012). It is suspected that leopards *Panthera pardus* were extirpated from Kibale prior to the 1970s, when tropical ecology research began in the forest (T. Struhsaker & T. Butynski, personal communication, 2015). This may leave African golden cats as the de facto apex predators in this community of small carnivores (Figure 1).

**Figure 1:**
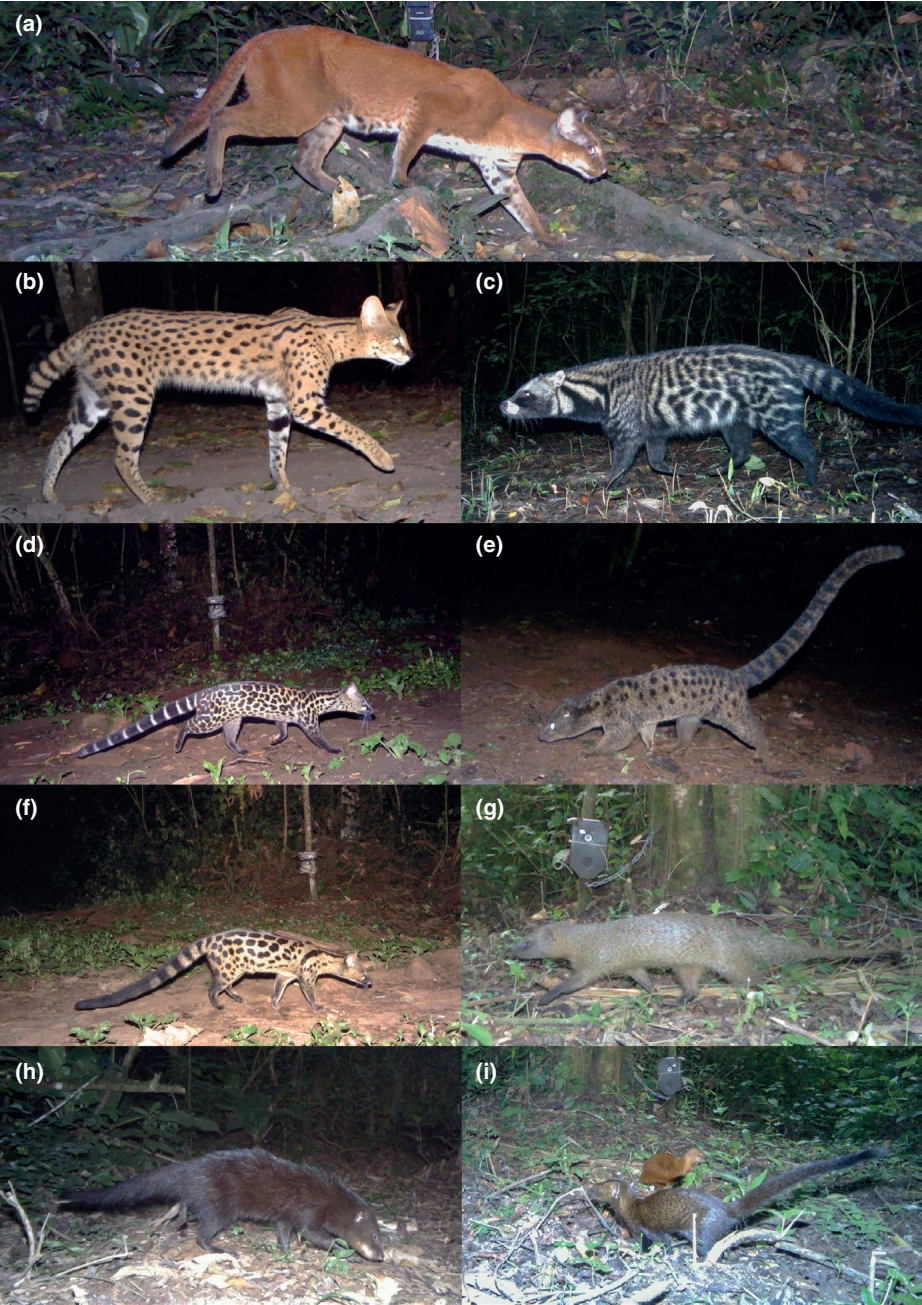
Nine species of small carnivores detected more than 30 times in Kibale National Park, Uganda, in 2013–2014. (a) African golden cat *Caracal aurata*, (b) Serval *Leptailurus serval*, (c) African civet *Civettictis civetta*, (d) Servaline genet *Genetta servalina*, (e) African palm civet *Nandinia binotata*, (f) Rusty‐spotted genet *Genetta maculata*, (g) Large gray mongoose *Herpestes ichneumon*, (h) Marsh mongoose *Atilax paludinosus*, and (i) Slender mongoose *Herpestes sanguineus*

### 2.2 Camera trap survey protocol

**Figure 2:**
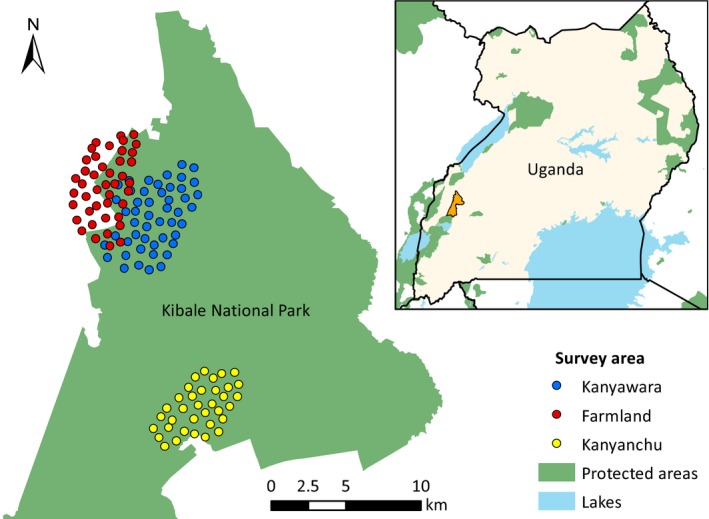
Location of three systematic camera trap surveys conducted in Kibale National Park (orange polygon in map inset), southwest Uganda, in 2013–2014

Between January 2013 and February 2014, we conducted three systematic camera trap surveys, in Kanyawara (49 stations), farmland adjacent to Kanyawara (43 stations), and Kanyanchu (35 stations; Figure 2). At each station, we placed one Pantheracam v4 camera trap, and a second digital (Panthera v4, Cuddeback IR, Stealthcam HD) or 35 mm film (Deercam, Camtracker) camera. Stations were spaced approximately 600–800 m apart. Cameras were placed in pairs, 1.5–2.5 m from active trails, and aimed 25 cm above the trail. Stations were operational for 76.6 (*SD* 13.8) days, with some variation due to trap failure and elephant *Loxodonta africana* damage. An attractant comprising a combination of meat, fruit and cologne (Calvin Klein Obsession for Men) was used at every station (Mills Fattebert, Hunter, & Slotow, 2019). We serviced cameras and refreshed attractants every 7–10 days. Surveys were conducted across both wet (March–May and September–November) and dry (June–August and December–February) seasons.

### 2.3 Niche partitioning in closely related species

We tested for differences in habitat specialization between closely related species by evaluating the influence of station‐level vegetation covariates on occupancy probability for each species. Around each camera station, we established a 30 m × 30 m plot and subdivided it into nine 10 m × 10 m subplots. We documented the species, diameter at breast height (DBH), and height of all trees with a DBH > 10 cm within each of these subplots. Canopy height was measured using a clinometer where tree density allowed clear line of sight, or with a rangefinder targeting the highest branches. Within each subplot, we established another randomly placed 3 m × 3 m plot where we counted the number of saplings with DBH < 10 cm and height > 1.5 m, and took a photograph of the canopy from the center point of the plot. Along the trails within the main plot, we measured trail width and took photographs of the undergrowth on each side of the trail at 5‐m intervals. Photographs were taken from 20 cm above the trail, at right angles to the trail, in both directions. Undergrowth density was measured from the photograph by calculating the proportion of a 1.0 m × 1.5 m red cloth covered by vegetation when placed 1.5 m from, and parallel to, the trail. We then calculated mean and standard deviation for all covariates at each station. We also hypothesized that tree diversity could influence habitat use and therefore calculated Shannon's Diversity Index (*H*), Fisher's Alpha, and Pielou's Evenness Index (Hill, 1973), for the diversity of large trees in each 30 m × 30 m plot (Table S1).

All statistical analyses in this study were conducted in the program (R Core Team, 2017), except where indicated. First, we identified collinear habitat variables using Pearson's correlation coefficient. When the absolute value of the correlation coefficient between two covariates was >0.70, the most significant covariate across all species was retained for analysis (Marinho, Bezerra, Antongiovanni, Fonseca, & Venticinque, 2018). Using the package *unmarked* (Fiske & Chandler, 2011), we constructed standard single‐species, single‐season occupancy models for each species based on zero‐inflated binomial models (MacKenzie et al., 2006). To improve model convergence, we collapsed detections into occasion lengths of 6 days (Farris et al., 2015). These models calculate the probability that a species occupies, or uses, a given site (Ψ). Even if a species occupies a site, factors, such as foraging habits, proximity of a camera station to home range center, proximity to food sources, and vegetation structure, can increase or reduce the chances that the species is photographed. These models allow us to calculate occupancy probability at sites where the target species was not detected by first modeling probability of detecting a species (p) at sites where the species was detected at least once. We tested mean and maximum trail width, mean and standard deviation of undergrowth density, and season (wet or dry), as possible detection covariates. We then univariately tested each vegetation covariate with the best detection model for each species and additively combined significant covariates in all possible combinations. We evaluated the resulting models using Akaike's information criterion adjusted for small sample sizes (AICc) (Burnham, Anderson, & Huyvaert, 2010). We assessed model fit by calculating the variance inflation factor (c‐hat) using the MacKenzie and Bailey goodness‐of‐fit test on the global (most complex) model (MacKenzie & Bailey, 2004). When overdispersion was indicated by a c‐hat value >1, we used this value to adjust the standard errors of the parameters and the AICc values, and ranked models using quasi‐AICc (QAICc) (Kéry & Royle, 2015). We model averaged covariates in all models with Δ(Q)AICc < 6, which have been shown to have some support (Richards, Whittingham, & Stephens, 2011). We then compared habitat preferences between pairs of closely related species (i.e., within cats, genets, or mongooses) to determine whether strong opposing habitat trends were present.

### 2.4 Activity pattern specialization

We modeled activity patterns for all focal species using package *circular* (Oliveira‐Santos, Zucco, & Agostinelli, 2013). Only independent captures, those that occurred at least 1 hr after the last detection of the same species, were used for analysis (Tobler, Carrillo‐Percastegui, Leite Pitman, Mares, & Powell, 2008). We used function *getBandWidth* from the *overlap* package to calculate the best smoothing parameter, *κ*, for each species (Ridout & Linkie, 2009). To describe the general activity period of each species, we used the function *modal.region.circular* to calculate the 95% activity isopleth. To identify core time periods of primary importance to each species, we calculated the activity period(s) representing the 50% activity isopleth.

To look for the patterns of opposing temporal specialization between closely related species, we used the function *totalvariation.circular* in R package *circular* to calculate the conditional overlap coefficient (OVL) in activity periods between species within each species family: felids, viverrids, and herpestids (Oliveira‐Santos et al., 2013). OVL is equivalent to ${\hat{\Delta }}_{1}$ described in Ridout and Linkie (2009). This measure is interpreted as the similarity between two distributions, whereby if OVL = 0, species have perfect dissimilarity, and if OVL = 1, the activity periods intersect fully (Oliveira‐Santos et al., 2013). We calculated OVL for both core activity periods (50% isopleths) and general activity periods (95% isopleths), of each species pair. To standardize the smoothing coefficient (*κ*), we used the mean of smoothing coefficients for all six species considered (Oliveira‐Santos et al., 2013).

### 2.5 Interference competition

In order to test for the effects of interference competition within the small carnivore community caused by the larger African golden cats, we constructed two species, single‐season occupancy models using the ΨBa/rBa parameterization in program PRESENCE v12.7 (Hines, 2006). These are an extension of the single‐season, single‐species models used above (MacKenzie et al., 2004). They directly estimate occupancy probability of two species, as well as their probabilities of occupancy and detection, conditional on the presence or absence of the other. We use the conditional two‐species model presented by Richmond et al. (2010), which allows covariates to be modeled on parameters of interest. In this model parameterization, species A represents the dominant species (African golden cat) and species B the subordinate species (smaller carnivores). These models evaluate the following parameters: the occupancy of species A (ΨA), the occupancy of species B given the presence of species A (ΨBA), the occupancy of species B given the absence of species A (ΨBa), the detection of species A given the absence of species B (pA), the detection of species B given the absence of species A (pB), the detection of species A given the presence of both species (rA), the detection of species B given the presence and detection of species A (rBA), and the detection of species B given the presence and nondetection of species A (rBa). We calculated conditional species interaction factors (SIF) for both occupancy and detection. These represent the likelihood with which species B occupies (Φ), or is detected (*δ*), at the same camera stations as species A (Richmond et al., 2010).

$$\Phi =\Psi A\;\Psi \mathrm{BA/}\Psi \mathrm{A(}\Psi A\;\Psi \mathrm{BA}+\left(1-\Psi A\right)\Psi \text{Ba)}$$

δ=rA rBA/rA(rA rBA+(1-rA)rBa)

A SIF value equal to 1 indicates independent occupancy or detection, a value <1 where confidence intervals do not overlap 1 indicates avoidance, and a value of >1 where confidence intervals do not overlap 1 indicates attraction (Richmond et al., 2010).

We constructed models where the occupancy and detection probabilities were independent of the presence and detection of the dominant species (ΨBA = ΨBa, pB = rBA = rBa), the presence of the dominant species influenced the presence (ΨBA ≠ ΨBa), or detection (pB ≠ rBA; pB ≠ rBa), of the subordinate, or the detection of the dominant species influenced the detection of the subordinate (rBA ≠ rBa). Apparent attraction and avoidance may be due to habitat preferences rather than interference competition. We therefore also constructed the same models using the most influential occupancy and detection habitat covariates from the single‐season occupancy models for each species. We ranked models using AIC (Burnham et al., 2010). For each pair, we report the most supported models (ΔAIC < 2.0).

To look for the possibility of facultative temporal specialization resulting from interference competition, we again used the function *totalvariation.circular* in R package *circular* to calculate the conditional overlap coefficient (OVL) in activity periods. We calculated OVL for both core activity periods (50% isopleths), and general activity periods (95% isopleths), between African golden cats and each of the other small carnivores. Finally, we compared activity patterns of each species at stations where African golden cats were detected, to their activity at stations where African golden cats were never detected.

## 3 RESULTS

### 3.1 Survey results

Over 9738 tap days, we detected 14 wild carnivore species (Table 2, Table S2). Most of these were captured infrequently (*n* < 50), or in very localized areas, and do not appear to be primary drivers of carnivore community dynamics in this particular ecosystem. Low detection rates may be the result of interference competition. However, occupancy models fail to converge with very low sample sizes. Therefore, we focused occupancy and co‐occupancy analyses on six carnivore species that were captured >50 times. We obtained a cumulative total of 3,917 photographs for these species: African golden cat, African civet *Civettictis civetta*, African palm civet *Nandinia binotata*, servaline genet *Genetta servalina*, rusty‐spotted genet *Genetta maculata*, and marsh mongoose *Atilax paludinosus* (Figure 1). Servals *Leptailurus serval*, large gray mongooses *Herpestes ichneumon*, and slender mongooses *Herpestes sanguineus* were captured 36, 37, and 48 times, respectively. Activity pattern modeling can accommodate lower sample sizes, so these species were included in the temporal analysis. No leopards were photographed during these surveys, nor was any leopard sign detected during camera placement and checks, supporting the assertion that leopards have been extirpated from Kibale.

**Table 2:** Carnivore species detected during three camera trap surveys in and near Kibale National Park, Uganda, in 2013–2014

Common name	Species	Photograph events	Capture rate	Naïve occupancy
Canidae				
Side‐striped jackal	*Canis adustus*	20	0.21	0.063
Felidae				
African golden cat	*Caracal aurata*	201	2.06	0.543
Serval	*Leptailurus serval*	36	0.37	0.189
Herpestidae				
Marsh mongoose	*Atilax paludinosus*	1,796	18.45	0.874
Alexander's cusimanse	*Crossarchus alexandri*	6	0.06	0.047
Slender mongoose	*Herpestes sanguineus*	48	0.49	0.150
Large gray mongoose	*Herpestes ichneumon*	37	0.38	0.165
Banded mongoose	*Mungos mungo*	11	0.11	0.031
Mustelidae				
Congo clawless otter	*Aonyx congicus*	17	0.17	0.063
Honey badger	*Mellivora capensis*	26	0.27	0.063
Nandiniidae				
African palm civet	*Nandinia binotata*	136	1.4	0.433
Viverridae				
African civet	*Civettictis civetta*	433	4.45	0.496
Rusty‐spotted genet	*Genetta maculata*	713	7.32	0.480
Servaline genet	*Genetta servalina*	520	5.34	0.732

Note

Total independent photographic events (>1 hr apart), capture rate (no. photographs/effective trap nights × 100), and naïve occupancy (% of stations where each species was detected) are presented for each species.

### 3.2 Covariate selection

Following correlation screening, three detection covariates (season, max trail width, and mean undergrowth density) and five occupancy covariates were retained: large stem density (with higher values representing older and/or less disturbed forest), small stem density (with higher values representing regenerating or disturbed forest), canopy height (representing forest age and type), canopy std. dev. (representing site heterogeneity—in this case the presence of bushy areas in the plot), and Shannon's diversity (representing species variation of large trees—with higher diversity being a proxy for undisturbed sites and possibly greater fruit and seed diversity, and therefore rodent presence and diversity).

### 3.3 Habitat specialization in closely related species

Wider trails increased detection probability of all species except African palm civets. Higher mean undergrowth density positively influenced detection probability of African golden cats, servaline genets, rusty‐spotted genets, and marsh mongooses. Season was in the best supported models for African golden cats, servaline genets, and marsh mongooses. Detection probability was higher during the dry season for all three species, though confidence intervals overlapped with detection during wet season (Table 3).

**Table 3:** Model averaged estimates of detection and occupancy covariates, with confidence intervals in parentheses, found in the best supported models for six small carnivores in Kibale National Park, Uganda

Covariates	African golden cat^a^	African civet^a^	African palm civet	Servaline genet	Rusty‐spotted genet	Marsh mongoose
Detection covariate						
Dry season^b^	0.11 (0.06 to 0.19)			0.31 (0.22 to 0.43)		0.66 (0.51 to 0.86)
Wet season^b^	0.04 (0.02 to 0.09)			0.20 (0.13 to 0.30)		0.50 (0.38 to 0.68)
Max trail width (mm)	**0.44 (0.28 to 0.60)**	**0.61 (0.44 to 0.77)**		**0.16 (0.03 to 0.28)**	**0.68 (0.53 to 0.83)**	0.10 (−0.01 to 0.21)
Undergrowth density (%)	**0.94 (0.04 to 1.84)**			**0.68 (0.14 to 1.22)**	**1.27 (0.60 to 1.95)**	**1.25 (0.83 to 1.68)**
Occupancy covariate						
Large stem density (no./ha)	**0.82 (0.64 to 2.85)**	0.02 (−0.45 to 0.65)	0.13 (−0.38 to 1.34)	**0.80 (0.26 to 1.58)**		−0.05 (−1.21 to 0.79)
Small stem density (no./ha)	1.44 (−0.13 to 4.05)	−0.03 (−0.63 to 0.34)	−0.12 (−1.40 to 0.46)			0.74 (−0.12 to 2.05)
Mean canopy height (m)		−0.03 (−0.67 to 0.40)	0.00 (−0.88 to 0.88)			0.01 (−0.87 to 0.93)
Mean understory height (m)		−0.04 (−0.64 to 0.31)	−0.01 (−0.60 to 0.50)			
Canopy cover std dev		0.04 (−3.03 to 3.57)	−0.22 (−5.57 to 3.31)			−1.13 (−7.41 to 1.83)
Canopy height std dev		−0.12 (−0.77 to 0.16)	0.30 (−0.14 to 1.47)			
Small stem std dev		−0.02 (−0.55 to 0.36)	−0.11 (−1.01 to 0.20)			
Undergrowth density std dev		−0.08 (−6.39 to 5.37)	0.33 (−5.10 to 8.68)			
Shannon's Diversity Index		0.00 (−0.50 to 0.52)	0.11 (−0.17 to 0.96)	**0.66 (0.22 to 1.49)**	**−0.79 (−1.34 to −0.27)**	0.30 (−0.08 to 1.18)
Pielou's Evenness Index		0.00 (−0.52 to 0.48)	0.13 (−0.25 to 1.20)			

Note

Significant differences between seasons are indicated by nonoverlapping confidence intervals between wet and dry season. To show the direction of the trend for the remaining continuous covariates, estimates were not converted to detection probability. Confidence intervals of significant continuous covariates do not overlap zero.

a The null occupancy model was the best supported model for both African civets and African palm civets.

b Model averaged estimates of the categorical season covariate were converted to detection probability to aid interpretation.

Increasing large stem density increased occupancy of African golden cats and servaline genets, and this term was in the best supported models of all species except rusty‐spotted genets. Higher small stem density increased occupancy of African golden cats. The diversity of large tree species (Shannon's *H*) increased occupancy probability of servaline genets, decreased that of rusty‐spotted genets, and was present in the best models (ΔAIC < 6) for all species except African golden cats. Confidence intervals of all covariate estimates crossed zero for African civets, African palm civets, and marsh mongooses, suggesting that none of the covariates tested in this study are influential in the occupancy of these species (Table 3, Table S3).

### 3.4 Activity pattern specialization in closely related species

**Figure 3:**
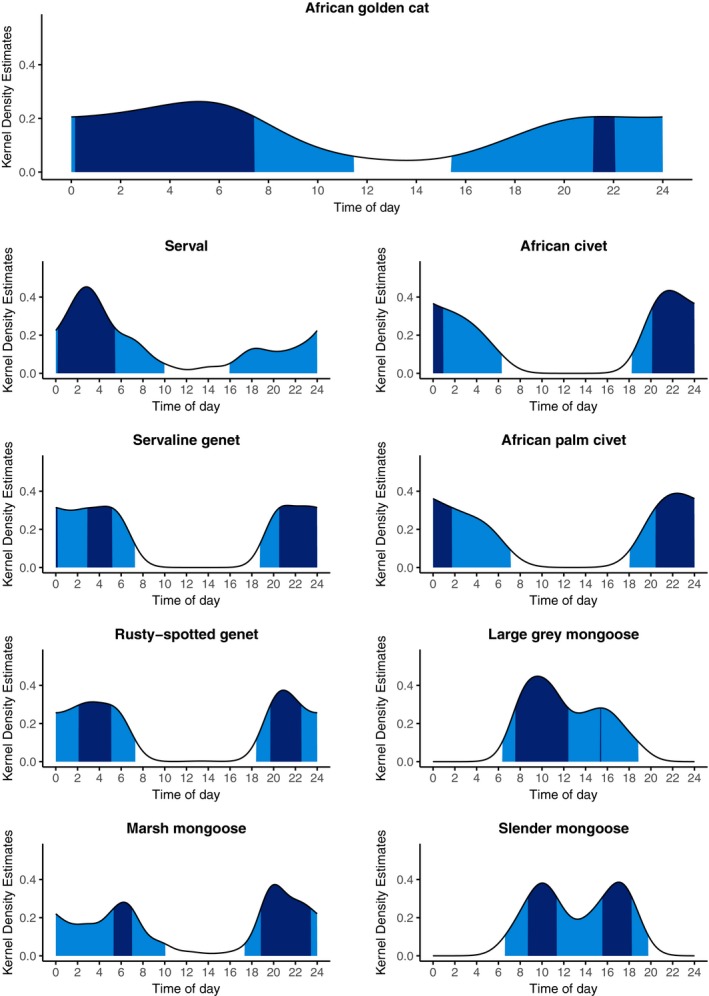
Diel activity patterns of nine small carnivores in Kibale National Park, Uganda, showing 50% core activity times (dark blue) and 95% general activity times (light blue)

Seven of our eight focal species were predominantly nocturnal with some crepuscular activity (Figure 3, Table S4). African palm civets, African civets, servaline genets, and rusty‐spotted genets, all became active at or just before dusk and ceased activity at or during dawn. The 95% activity isopleths for African golden cats, servals, and marsh mongooses, all indicate a greater probability of diurnal activity than those of the other nocturnal species in this study. The large gray mongoose and slender mongoose were the only diurnal species. Mean 50% core activity period for African golden cats was the longest (8.19 hr); the mean core activity period for all other species was 5.54 hr (*SD* 0.58). Five of the eight species showed a peak in activity for several hours just after dusk (African civet, African palm civet, servaline genet, rusty‐spotted genet, marsh mongoose). Both genet species and marsh mongoose had bimodal core activity periods, with a lull in activity in the middle of the night. The African golden cat's core activity period began later than the other nocturnal species. Core activity of servals was nocturnal, occurring after midnight; large gray mongooses were primarily active in the morning; and slender mongooses had two distinct activity peaks in the morning and evening. However, servals, large gray mongooses, and slender mongooses were photographed relatively infrequently in this study, so temporal modeling should be interpreted with caution.

**Figure 4:**
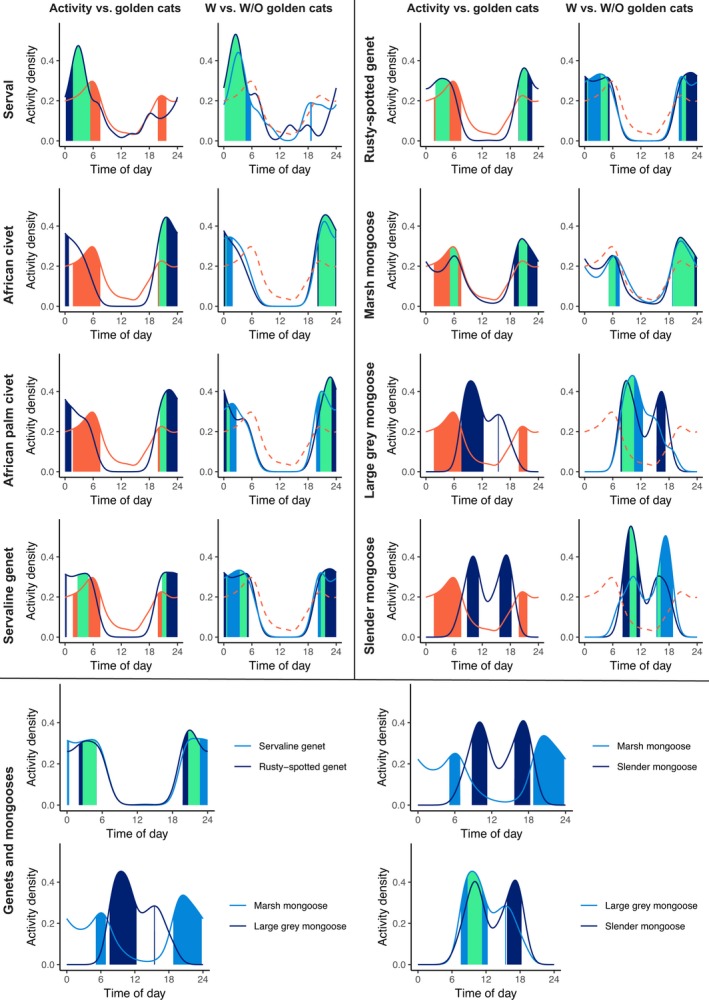
First and third columns: A comparison of overlap (indicated in green) between 50% core activity patterns of African golden cats (orange) and eight other small carnivore species (dark blue). Second and fourth columns: A comparison of overlap (green) between 50% core activity patterns of eight small carnivore species in the presence of (dark blue) and absence of (light blue) African golden cats. Bottom four plots: Overlap between 50% core activity patterns (green) of related species (genets and mongooses). When no overlap is present, only the respective core activity periods are displayed. All data were collected in Kibale National Park, Uganda, in 2013–2014

Opposing patterns in closely related species were only seen in the mongooses, which had strongly segregated activity patterns between two diurnal and one nocturnal species (Table 4, Figure 4). The cats had similar general activity patterns and moderate overlap of 50% core activity periods, and both general and core activity periods of the genets overlapped extensively.

**Table 4:** Coefficient of overlap (OVL) of both core and general activity periods between five pairs of closely related carnivore species in Kibale National Park, Uganda, in 2013–2014

Species	Core (50% isopleth)	General (95% isopleth)
African golden cat versus serval	0.47	0.80
Servaline genet versus rusty‐spotted genet	0.71	0.95
Marsh mongoose versus large gray mongoose	0.00	0.20
Marsh mongoose versus slender mongoose	0.00	0.22
Large gray mongoose versus slender mongoose	0.49	0.83

### 3.5 Interference competition: spatial displacement

For all species, the highest ranked models included habitat covariates from the best single‐species single‐season occupancy models (Table S5). We found no evidence that interference competition by African golden cats was causing spatial displacement of other carnivores. In fact, the occupancy SIF (Φ) was positive for African civets and rusty‐spotted genets, indicating co‐occupancy of locations more frequently than predicted (Table 5). However, both African civets and African palm civets had lower detection probabilities when African golden cats were present and detected (*δ* < 1), suggesting reactive avoidance. Conversely, both genet species had higher detection probabilities when African golden cats were present and detected (Table 5).

**Table 5:** Species interaction factors (SIF) for co‐occurrence (Φ) and codetection (*δ*) between African golden cats and each of four small carnivore species in Kibale National Park, Uganda, in 2013–2014

The effect of African golden cat on	Occupancy SIF				Detection SIF			
	Φ	SE	LCl	UCl	*δ*	SE	LCl	UCl
African civet	1.15	0.02	1.12	1.19	0.87	0.04	0.80	0.94
African palm civet	–	–	–	–	0.04	0.04	−0.04	0.11
Rusty‐spotted genet	1.09	0.02	1.06	1.13	1.55	0.04	1.47	1.63
Servaline genet	0.97	0.10	0.78	1.16	1.24	0.01	1.22	1.26

Note

Both SIF measures are presented with standard errors and confidence intervals. Avoidance is indicated if the SIF is below 1 and attraction if it is above 1. Significant interactions are indicated when confidence intervals do not overlap 1. The best model for African palm civets did not include an interaction effect on the occupancy parameter.

### 3.6 Interference competition: temporal overlap

There were high coefficients of overlap for 95% activity periods between African golden cats and all other species except the diurnal large gray mongoose and slender mongoose (Table 6, Figure 4). Overlap of 50% core activity periods was low for African civets and African palm civets, but activity patterns were similar at stations with and without African golden cats. Large gray mongooses had an evening activity peak at stations with African golden cats, and slender mongooses had an evening activity peak at stations without African golden cats.

**Table 6:** Coefficient of overlap (OVL) in activity patterns between African golden cats and each of six carnivore species in Kibale National Park, Uganda, in 2013–2014

Species	Overlap with African golden cat activity period		Stations with versus without African golden cats	
	Core (50%)	General (95%)	Core (50%)	General (95%)
Serval	0.47	0.80	0.81	0.78
African civet	**0.18**	0.66	0.76	0.92
African palm civet	**0.16**	0.70	0.56	0.89
Servaline genet	0.42	0.77	0.43	0.95
Rusty‐spotted genet	0.60	0.78	0.61	0.88
Marsh mongoose	0.44	0.87	0.87	0.91
Large gray mongoose	**0.00**	**0.25**	0.54	0.82
Slender mongoose	**0.00**	**0.26**	**0.34**	**0.78**

Note

Left: overlap between the respective species' activity patterns. Right: comparison of overlaps between activity patterns of each species at stations occupied by (with) and not occupied by (without) African golden cats. Values in bold indicate low overlap with African golden cat activity period.

## 4 DISCUSSION

We found multiple drivers structuring the studied carnivore community. Niche partitioning between closely related species (limiting similarity) and weak interference competition play a role in the observed community membership, and niche selection. Fourteen wild small carnivore species were detected during this survey, but only six were captured frequently enough to include in our spatial analyses. Low detection rates of other species probably stem from a variety of drivers. Habitat preference is certainly an important cause. For example, servals and large gray mongooses prefer open habitats, and Congo clawless otters *Aonyx congicus* prefer wetlands (Kingdon & Hoffmann, 2013). However, it is possible that populations of some species were suppressed due to interference competition with African golden cats or other predators (Gehrt, Wilson, Brown, & Anchor, 2013; Levi & Wilmers, 2012).

### 4.1 Niche specialization

We found that the African golden cat and servaline genet were forest specialists and the African civet was a habitat generalist, which confirms current understanding of these species' ecology (Ray, 2013; Ray & Butynski, 2013; Van Rompaey & Colyn, 2013). Though African civets overlap in activity patterns, diet, and habitat with most of the other carnivores, they are omnivorous and exploit both open and forested areas, allowing them to divide competition among all specialists, and exploit resources when and where they are available. Somewhat unexpectedly, occupancy of the primarily arboreal African palm civet was not influenced by measures of increased forest cover. While marsh mongooses associate closely with waterways in other parts of their range (Baker, 1987; Ray, 1997), we did not find this to be true in Kibale, where they were captured at 87% of camera stations, even at locations that were not adjacent to streams. Their specialization in riparian habitats elsewhere may be a facultative response to competition, rather than an obligate specialization resulting from niche partitioning between closely related species. This could also mean that habitat preferences of marsh mongooses are not necessarily consistent across their range. This supports recent evidence that the influence of covariates on occupancy models varies geographically (Rich et al., 2017). It also highlights the difficulty in understanding obligate (what they must use), preferred (what they want to use), and facultative (what they are forced to use), resource use within species.

All focal species except the large gray mongoose and slender mongoose were nocturnal, which again confirms our current understanding of most of these species' ecology (Hunter & Barrett, 2019; Kingdon, 2015). Both genet species and marsh mongoose showed a slight reduction in activity in the early hours of the day, followed by a second burst of activity before dawn. Elsewhere in their range, rusty‐spotted genets have been reported to be most active during the first part of the night followed by a short rest period (Angelici & Gaubert, 2013). While we have identified apparent nocturnal specializations in this study, this does not imply that the corresponding species are specialists across their range. The African golden cat, for example, is cathemeral in other forests where leopards persist (Bahaa‐el‐din, 2015), and is, therefore, an activity pattern generalist species, with specialist individuals (Alves et al., 2017). This emphasizes the importance of studying species in a variety of ecosystems.

Most interestingly, the diversity of mature tree species was present in the best supported models for all species except African golden cats. While forest removal may extirpate some of these carnivore species, a reduction in tree diversity through selective logging may also influence carnivore community assembly and relative abundances. In this way, simplification of forest tree biodiversity may precipitate reductions in mammal biodiversity. Increased plant diversity is thought to positively affect top‐down control by increasing predation rates, as has been suggested for bird–insect interactions (Barbaro et al., 2017; Yang et al., 2017), and is associated with higher arthropod diversity (Haddad et al., 2009). These mechanisms may similarly increase prey diversity and predation rates of insectivorous carnivores, such as servaline genets (Van Rompaey & Colyn, 2013). Ultimately, the underlying drivers for increased use of sites with high tree diversity by forest specialists are not necessarily straightforward. Tree diversity may be associated with increased hunting success, increased prey diversity, the presence of rare or preferred prey, or preferred resting areas in more complex forest structures. Further study is required to parse these potential factors. It may therefore be important to include measures of plant or tree species diversity in species distribution models for small carnivores, particularly semi‐arboreal species (Fricker, Wolf, Saatchi, & Gillespie, 2015; Rocchini et al., 2016).

### 4.2 Niche partitioning in closely related species

We acknowledge that there are complex processes influencing community assembly, and that the most important processes driving community assembly often change over time, creating a “ghost of competition past,” which makes it difficult to definitively identify causality in currently observed assemblies (Chesson, 2000; Connell, 1980; Pianka, 1981; Vergnon et al., 2012). Here, however, we took a relatively simplistic approach as a starting point to understanding this relatively unstudied carnivore community.

The theory of limiting similarity remains contentious, but we found some evidence of this process in Kibale, where the most closely related species partitioned either habitat or activity periods. The felids were both primarily nocturnal, but had less than 50% overlap in core activity periods. Servals were also only captured in or near grassland and bush, therefore partitioning habitat use with the forest‐dependent African golden cat. The genet species had similar activity patterns, but servaline genets preferred more diverse (less disturbed) forest with more saplings, while rusty‐spotted genets preferred less diverse locations with lower sapling density, including monocultures of eucalyptus and pine, suggesting these species strongly partition habitat use. The mongooses had strongly opposing activity patterns with very little overlap. Additionally, though large gray mongooses were not captured frequently enough to include them in single‐species occupancy models, they were captured only in open habitats, while marsh mongoose was associated with higher densities of saplings, as is found in secondary forest, suggesting some habitat partitioning as well (Guariguata, Chazdon, Denslow, Dupuy, & Anderson, 1997).

### 4.3 Interference competition and facultative specialization

While the removal of an apex predator can result in the emergence of a secondary apex predator (Prugh et al., 2009; Ritchie & Johnson, 2009), our results suggest that African golden cats are not significantly structuring the habitat niches of the other small carnivores in Kibale through interference competition. First, they did not negatively influence the occupancy of any of the five other most photographed species in Kibale. In fact, we found that they had a weak positive influence on the occupancy probability of marsh mongooses and rusty‐spotted genets. Additionally, both genet species had higher detection probabilities where African golden cats were also detected. While there are several plausible explanations for this, shared prey species is the most likely reason (Kingdon & Hoffmann, 2013).

While all of these species prey on rodents and therefore have considerable dietary overlap (Kingdon & Hoffmann, 2013), we acknowledge that food may be sufficiently abundant that interference competition is relatively low. Additionally, there may be some reactive, ad hoc avoidance strategies employed at a fine spatial scale by subordinate carnivores when they detect an African golden cat, as observed in other carnivore communities (Broekhuis, Cozzi, Valeix, McNutt, & Macdonald, 2013; López‐Bao, Mattisson, Persson, Aronsson, & Andrén, 2016; Vanak et al., 2013). Additionally, their arboreal habits may allow African palm civets to partition habitat vertically, thereby reducing interference competition in a way that we were not able to test (Charles‐Dominique, 1978; Van Rompaey & Ray, 2013). We stress that SIF is a correlative measure. We have shown interesting patterns that suggest avoidance and facilitation. These results should be regarded as a guide to understanding interactions, rather than proof of an underlying process.

Although there was no clear partitioning of diel activity when considering general activity periods, there was some partitioning of core activity periods. While African golden cats focused most of their activity from late evening until dawn, the other species focused most of their core activity between dusk and late evening, or in the early morning. This lull in activity around the time African golden cats become active may be a strategy to avoid interference competition, but it may also represent the fundamental preferred activity period of African civets and African palm civets (Ray, 2013; Van Rompaey & Colyn, 2013; Van Rompaey & Ray, 2013). However, when combined with lower detection probabilities, this may suggest that African civets and African palm civets reactively avoid stations (or trails) when they detect African golden cats in the area. The diurnal mongooses have differing activity patterns between stations with and without African golden cats. Because African golden cats are nocturnal and therefore are unlikely to directly interact with these diurnal species on a regular basis, this difference in activity within these mongoose species may be linked to other drivers, such as human presence or forest cover.

We must emphasize that, due to the nature of camera trap data, these detections occurred randomly, with some detection soon after an African golden cat detection, and some occurring months after a golden cat detection. Therefore, these analyses only had the power to detect broad changes in activity patterns that represented predictive avoidance of periods of high encounter risk. Again, individuals of subordinate species may be employing a more reactive strategy. Despite these limitations, we have shown that, where they are not using different habitats, they are coexisting with a high degree of overlap in activity patterns. We look forward to future studies that investigate fine‐scale coexistence strategies.

## 5 CONCLUSIONS

Here, there was evidence for temporal and/or spatial separation between related species. In particular, we recorded several obligate forest specialists, which are typically sensitive to localized extirpation through deforestation (Barlow et al., 2016; Ochoa‐Quintero, Gardner, Rosa, Barros Ferraz, & Sutherland, 2015; Prist, Michalski, & Metzger, 2012). This could have impacts on carnivore community dynamics, by altering spatial and diel activity patterns. As with most ecosystems, perturbations will negatively impact some species, particularly specialists who utilize the affected resources, and allow others, mainly generalists, to expand their niches. In order to maintain the observed community assemblage and its functionality, we must maintain diverse ecosystems, with healthy habitats required by habitat specialists.

We found little evidence for interference competition between African golden cats and other small carnivores. Competition is complex, and there may be other factors perpetuating the coexistence of these species. In the absence of leopards, it is possible that golden cats have undergone release enabling them to better exploit larger prey, and reduce interference competition for smaller prey (i.e. Rodentia) with other species. If this tropical forest is representative, abundant food sources may create a system that is driven by intraspecific competition, rather than interspecific competition (Chesson, 2000).

If interference competition does not strongly influence assembly in these communities, we may not need to worry about abrupt community restructuring seen elsewhere (Levi & Wilmers, 2012; Terborgh & Estes, 2010). Instead, we can focus on mitigating external pressures, such as bushmeat hunting and habitat destruction (Haddad et al., 2015; Ripple et al., 2016). As tropical forests are cut down in the face of rapid development, understanding the mechanisms underpinning community assembly is essential. This information will allow us to predict how disturbances and development will impact assemblages, and how to potentially mitigate degradation.

## CONFLICT OF INTEREST

None declared.

## AUTHORS' CONTRIBUTIONS

DM and RS conceived the ideas and designed methodology; DM and SI collected the data; DM analyzed the data; DM led the writing of the manuscript. All authors contributed critically to the drafts and gave final approval for publication.

## Data Availability

Dryad Digital Repository provisional DOI: https://doi.org/10.5061/dryad.6fq3qv7.
