# Supplementary material for: Competition and specialization in an African forest carnivore community
Source: Ecol Evol. 2019 Aug 27;9(18):10092–108. doi: 10.1002/ece3.5391 (PMC6787825; doi:10.1002/ece3.5391)
Supplement: Supplementary file 1 [file ECE3-9-10092-s001.pdf]

Supplementary Information

Mills et al. 2019

Competition and specialisation in an African forest carnivore community.

Supplementary Information 1. Station covariates recorded within 30 m × 30 m plots around each camera trap station during camera surveys in Kibale National Park, Uganda, in 2013–2014.

Measure	Abbreviation	Units	Description
**Large trees (DBH > 10 cm)**			
Large stem density	lrgStemDens	count/ha	Raw count of large trees
Basal area	BA	m^2/ha	Total area occupied by trees
Mean canopy height	mean.cnpy.hgt		
Max canopy height	max.cnpy.hgt	m	Canopy height above random small tree plots
SD canopy height	sd.cnpy.hgt		
Mean tree height	mean.tree.hgt		
Max tree height	max.tree.hgt	m	Individual tree heights
SD tree height	sd.tree.hgt		
Mean large tree diameter	mean.dia		
Max large tree diameter	max.dia	cm	Diameter at breast height
SD large tree diameter	sd.dia		
SD plot canopy height	sd.hgt.plot	m	Variation between subplots
SD plot tree diameter	sd.dia.plot		
**Small trees (DBH < 10 cm)**			
Small stem density	smStemDens	no./ha	Raw count of small trees
SD of small stem counts	sd.sm.stem	no.	Variation in small stem count
**Canopy cover**			
Mean canopy cover	cnpy.mean		
Max canopy cover	cnpy.max	%	Canopy cover above small tree plots
Min canopy cover	cnpy.min		
SD canopy cover	cnpy.sd		
Camera site canopy cover	cnpy.cam	%	Canopy cover above camera
**Undergrowth**			
Mean undergrowth density	ugd.mean		
Max undergrowth density	ugd.max	%	Percent of red cloth hidden by vegetation
Min undergrowth density	ugd.min		
SD undergrowth density	udg.sd		
**Large tree species diversity and evenness**			
Fisher's Alpha	fisher.alpha		
Shannon's Diversity Index	shanon		
Pielou's Evenness Index	pielous		
**Trail width**			
Mean trail width	trl_w_mean	cm	Width of all trails passing in front of the camera
Max trail width	trl_w_all		
Season	season	wet / dry	Whether the occasion fell in wet or dry season

Supplementary Information 2. Body measurements of all wild carnivore species detected in Kibale National Park, Uganda, 2013–2014.

Species	Common	Weight	mean HB
**Canidae**			
Canis adustus	Side-striped jackal	♀ 6.2–10 kg, ♂ 5.9–12 kg	75 cm
**Felidae**			
Caracal aurata	African golden cat	♀ 5.3–8.2 kg, ♂ 8–16 kg	80 cm
Leptailurus serval	Serval	♀ 6–12.5 kg, ♂ 7.9–18 kg	84 cm
**Herpestidae**			
Atilax paludinosus	Marsh mongoose	2.4–4.1 kg	50 cm
Crossarchus alexandri	Alexander’s cusimanse	1–2 kg	45 cm
Herpestes sanguineus	Slender mongoose	0.37–0.79 kg	33 cm
Herpestes ichneumon	Large grey mongoose	♀ 2.2–4 kg, ♂ 2.6–4.1 kg	58 cm
Mungos mungo	Banded monogoose	0.9–1.9 kg	37 cm
**Mustelidae**			
Aonyx congicus	Congo clawless otter	15–25 kg	84 cm
Mellivora capensis	Honey badger	♀ 6.2–13 kg, ♂ 7.7–14.5 kg	69 cm
**Nandiniidae**			
Nandinia binotata	African palm civet	1.2–3 kg	50 cm
**Viverridae**			
Civettictis civetta	African civet	7–20 kg	79 cm
Genetta maculata	Rusty-spotted genet	♀ 1.3–2.5 kg, ♂ 1.4–3.2 kg	49 cm
Genetta servalina	Servaline genet	2.3–3 kg	50 cm

Sources:

Hunter, L. & Barrett, P. (2011) Carnivores of the World. Princeton University Press, Princeton, NJ.

Jones, K.E., Bielby, J., Cardillo, M., Fritz, S.A., O’Dell, J., Orme, C.D.L., Safi, K., Sechrest, W., Boakes, E.H. & Carbone, C. (2009) PanTHERIA: a species-level database of life history, ecology, and geography of extant and recently extinct mammals. Ecology, 90, 2648-2648.

Kingdon, J. & Hoffmann, M. (2013) Mammals of Africa. Volume V: carnivores, pangolins, equids and rhinoceroses. Bloomsbury Publishing, London.

Supplementary Information 3. Ranked single-species single-season occupancy models for six carnivore species in Kibale National Park, Uganda. By convention, models with Δ(Q)AIC < 2 are equally supported, while Models with Δ(Q)AIC < 6 have some support (Richards, Whittingham & Stephens 2011). Therefore, we present equally supported occupancy models (plus the null occupancy model) for reference, while we use those with Δ(Q)AIC < 6 for model averaging (Table 3 in the main text). QAICc was used when overdispersion was indicated by a c-hat value > 1. Covariate abbreviations are available in Supplementary Information 1.

Model	DF	logLik	(Q)AICc	Δ(Q)AICc	weight	c-hat
**African golden cat**						
psi(smStemDens), p(season + trail.max + ugd.mean)	6	-451.48	915.75	0.00	0.530	1.00
psi(lrgStemDens), p(season + trail.max + ugd.mean)	6	-452.18	917.15	1.40	0.263	1.00
psi(lrgStemDens + smStemDens), p(season + trail.max + ugd.mean)	7	-451.27	917.63	1.88	0.207	1.00
psi(.), p(season + trail.max + ugd.mean)	5	-465.15	940.86	25.11	0.000	1.00
**African civet**						
psi.(.), p(trail.max)	3	-459.40	925.03	0.00	0.034	1.00
psi.(cnpyhgt5.sd), p(trail.max)	4	-458.50	925.37	0.34	0.029	1.00
psi.(cnpyunder.mean), p(trail.max)	4	-459.10	926.59	1.56	0.016	1.00
psi.(cnpyhgt5.mean), p(trail.max)	4	-459.19	926.77	1.74	0.014	1.00
psi.(smStemDens), p(trail.max)	4	-459.26	926.89	1.86	0.013	1.00
psi.(sm.stem.sd), p(trail.max)	4	-459.29	926.96	1.93	0.013	1.00
**African palm civet**						
psi.(.), p(.)	2	-351.40	281.83	0.00	0.021	2.55
psi.(cnpyhgt5.sd), p(.)	3	-348.72	281.88	0.05	0.020	2.55
psi.(Shannon), p(.)	3	-349.76	282.69	0.87	0.014	2.55
psi.(pielous), p(.)	3	-349.95	282.84	1.01	0.013	2.55
psi.(cnpyhgt5.sd + sm.stem.sd), p(.)	4	-347.21	282.89	1.06	0.012	2.55
psi.(sm.stem.sd), p(.)	3	-350.55	283.31	1.49	0.010	2.55
psi.(cnpyhgt5.sd + Shannon), p(.)	4	-347.79	283.35	1.52	0.010	2.55

Model	DF	logLik	(Q)AICc	Δ(Q)AICc	weight	c-hat
psi.(lrgStemDens), p(.)	3	-350.70	283.43	1.60	0.009	2.55
psi.(cnpyhgt5.sd + pielous), p(.)	4	-347.96	283.48	1.65	0.009	2.55
psi.(cnpyhgt5.sd + smStemDens), p(.)	4	-348.22	283.68	1.85	0.008	2.55
psi.(cnpyhgt5.mean), p(.)	3	-351.03	283.69	1.86	0.008	2.55
psi.(smStemDens), p(.)	3	-351.04	283.70	1.87	0.008	2.55
psi.(cnpyhgt5.sd + lrgStemDens), p(.)	4	-348.25	283.70	1.88	0.008	2.55
**Servaline genet**						
psi.(lrgStemDens + Shannon), p(season + trail.max + ugd.mean)	7	-741.68	1012.95	0.00	0.650	1.49
psi.(.), p(season + trail.max + ugd.mean)	5	-753.84	1024.67	11.72	0.002	1.49
**Rusty-spotted genet**						
psi.(Shannon), p(trail.mean + ugd.mean)	5	-462.35	831.12	0.00	0.979	1.13
psi.(.), p(trail.mean + ugd.mean)	4	-468.30	839.42	8.30	0.015	1.13
**Marsh mongoose**						
psi.(smStemDens), p(season + trail.mean + ugd.mean)	6	-945.75	1904.31	0.00	0.150	1.00
psi.(Shannon + smStemDens), p(season + trail.mean + ugd.mean)	7	-944.78	1904.64	0.33	0.127	1.00
psi.(cnpy.sd + Shannon), p(season + trail.mean + ugd.mean)	7	-945.35	1905.77	1.46	0.072	1.00
psi.(cnpy.sd + Shannon + smStemDens), p(season + trail.mean + ugd.mean)	8	-944.27	1905.94	1.63	0.066	1.00
psi.(cnpy.sd + smStemDens), p(season + trail.mean + ugd.mean)	7	-945.49	1906.05	1.74	0.063	1.00
psi.(.), p(season + trail.mean + ugd.mean)	5	-950.59	1911.75	7.44	0.004	1.00

Supplementary Information 4. Activity period (95% isopleth) and core activity period (50% isopleth) of nine carnivore species in Kibale National Park, Uganda in 2013–2014. The best smoothing parameter (κ) was individually calculated for each species.

Species	Independent photographs	K	Activity range (hour intervals)	
			Kernel 50%	Kernel 95%
African golden cat	201	2.7	21:09–22:04<br>00:09–07:26	15:24–11:28
Serval	36	8.2	00:09–05:27	15:56–09:58
African civet	433	8.4	20:06–00:56	18:14–06:18
African palm civet	137	6.0	20:09–01:48	18:02–07:07
Servaline genet	520	11.9	20:30–00:10<br>02:53–05:11	18:43–07:16
Rusty-spotted genet	713	11.8	19:42–22:33<br>02:05–05:05	18:23–07:17
Marsh mongoose	1796	21.3	18:49–23:25<br>05:18–07:00	17:20–10:03
Large grey mongoose	37	9.1	07:32–12:25<br>15:19–15:26	06:20–18:51
Slender mongoose	48	8.0	08:42–11:21<br>15:32–18:14	06:35–19:45

Supplementary Information 5a. Co-occurrence occupancy models describing interactions between African civets (species B) and African golden cats (species A). The best models for all species included the most significant covariates from single-species single-season occupancy models. Covariate abbreviations are explained in Supplementary Information 1. Parameters are explained in the Methods. Models are constructed to test whether parameters of interest equal (=) or not equal (!=) to each other. The best model was selected based on AIC.

**African golden cat – African civet models**	**AIC**	**ΔAIC**	**AIC weight**	**Model Lik.**	**no. param.**	**-2LogLik**
(ΨA)cnpy+smstem, (ΨBA != ΨBa), (pA = rA, pB != rBa != rBA)trail+ugd	3890.63	0	0.6341	1	17	3856.63
(ΨA)cnpy+smstem, (ΨBA = ΨBa), (pA = rA, pB != rBa != rBA)trail+ugd	3891.73	1.1	0.3659	0.5769	16	3859.73
(ΨA)cnpy+smstem, (ΨBA = ΨBa), (pA = rA, pB != rBa = rBA)trail+ugd	3969.07	78.44	0	0	13	3943.07
(ΨA)cnpy+smstem, (ΨBA != ΨBa), (pA = rA, pB != rBa = rBA)trail+ugd	4022.31	131.68	0	0	14	3994.31
(ΨA)cnpy+smstem, (ΨBA != ΨBa), (pA = rA, pB = rBa = rBA)trail+ugd	4098.75	208.12	0	0	11	4076.75
(ΨA)cnpy+smstem, (ΨBA = ΨBa), (pA = rA, pB = rBa = rBA)trail+ugd	4099.84	209.21	0	0	10	4079.84
ΨA, ΨBA != ΨBa, pA = rA, pB != rBa != rBA	4100.31	209.68	0	0	7	4086.31
(ΨA)cnpy+smstem, (ΨBA != ΨBa), (pA = rA, pB = rBa != rBA)trail+ugd	4101.7	211.07	0	0	14	4073.7
(ΨA)cnpy+smstem, (ΨBA = ΨBa), (pA = rA, pB = rBa != rBA)trail+ugd	4102.8	212.17	0	0	13	4076.8
ΨA, ΨBA = ΨBa, pA = rA, pB != rBa != rBA	4122.61	231.98	0	0	6	4110.61
ΨA, ΨBA != ΨBa, pA = rA, pB = rBa = rBA	4253.76	363.13	0	0	5	4243.76
ΨA, ΨBA != ΨBa, pA = rA, pB = rBa != rBA	4255.75	365.12	0	0	6	4243.75
ΨA, ΨBA = ΨBa, pA = rA, pB = rBa = rBA	4256.45	365.82	0	0	4	4248.45
ΨA, ΨBA = ΨBa, pA = rA, pB = rBa != rBA	4258.45	367.82	0	0	5	4248.45
ΨA, ΨBA = ΨBa, pA = rA, pB != rBa = rBA	4314.87	424.24	0	0	5	4304.87
ΨA, ΨBA != ΨBa, pA = rA, pB != rBa = rBA	4316.87	426.24	0	0	6	4304.87

Supplementary Information 5b. Co-occurrence occupancy models describing interactions between African palm civets (species B) and African golden cats (species A). The best models for all species included the most significant covariates from single-species single-season occupancy models. Covariate abbreviations are explained in Supplementary Information 1. Parameters are explained in the Methods. Models are constructed to test whether parameters of interest equal (=) or not equal (!=) to each other. The best model was selected based on AIC.

**African golden cat – African palm civet models**	**AIC**	**ΔAIC**	**AIC weight**	**Model Lik.**	**no. param.**	**-2LogLik**
(ΨA)cnpy+smstem, (ΨBA = ΨBa)ch5sd, (pA = rA, pB = rBa != rBA)trail + ugd	2812.13	0	0.4113	1	14	2784.13
(ΨA)cnpy+smstem, (ΨBA = ΨBa)ch5sd, (pA = rA, pB = rBa = rBA)trail + ugd	2812.34	0.21	0.3703	0.9003	11	2790.34
(ΨA)cnpy+smstem, (ΨBA != ΨBa)ch5sd, (pA = rA, pB = rBa = rBA)trail + ugd	2813.43	1.3	0.2147	0.522	13	2787.43
(ΨA)cnpy+smstem, (ΨBA != ΨBa)ch5sd, (pA = rA, pB = rBa != rBA)trail + ugd	2821.58	9.45	0.0036	0.0089	16	2789.58
(ΨA)cnpy+smstem, (ΨBA != ΨBa)ch5sd, (pA = rA, pB != rBa = rBA)trail + ugd	2835.57	23.44	0	0	16	2803.57
(ΨA)cnpy+smstem, (ΨBA = ΨBa)ch5sd, (pA = rA, pB != rBa = rBA)trail + ugd	2837.68	25.55	0	0	14	2809.68
(ΨA)cnpy+smstem, (ΨBA = ΨBa)ch5sd, (pA = rA, pB != rBa != rBA)trail + ugd	2851.76	39.63	0	0	17	2817.76
(ΨA)cnpy+smstem, (ΨBA != ΨBa)ch5sd, (pA = rA, pB != rBa != rBA)trail + ugd	2852.43	40.3	0	0	19	2814.43
ΨA, ΨBA = ΨBa, pA = rA, pB != rBa = rBA	2894.02	81.89	0	0	5	2884.02
ΨA, ΨBA = ΨBa, pA = rA, pB = rBa = rBA	2895.02	82.89	0	0	4	2887.02
ΨA, ΨBA != ΨBa, pA = rA, pB != rBa = rBA	2895.25	83.12	0	0	6	2883.25
ΨA, ΨBA = ΨBa, pA = rA, pB != rBa != rBA	2895.4	83.27	0	0	6	2883.4
ΨA, ΨBA = ΨBa, pA = rA, pB = rBa != rBA	2896.13	84	0	0	5	2886.13
ΨA, ΨBA != ΨBa, pA = rA, pB = rBa = rBA	2896.98	84.85	0	0	5	2886.98
ΨA, ΨBA != ΨBa, pA = rA, pB = rBa != rBA	2898.08	85.95	0	0	6	2886.08
ΨA, ΨBA != ΨBa, pA = rA, pB != rBa != rBA	2912.1	99.97	0	0	7	2898.1

Supplementary Information 5c. Co-occurrence occupancy models describing interactions between servaline genets (species B) and African golden cats (species A). The best models for all species included the most significant covariates from single-species single-season occupancy models. Covariate abbreviations are explained in Supplementary Information 1. Parameters are explained in the Methods. Models are constructed to test whether parameters of interest equal (=) or not equal (!=) to each other. The best model was selected based on AIC.

**African golden cat – servaline genet models**	**AIC**	**ΔAIC**	**AIC weight**	**Model Lik.**	**no. param.**	**-2LogLik**
(ΨA)cnpy+smstem, (ΨBA != ΨBa)lgstem+shannon, (pA = rA, pB != rBa != rBA)trail+ugd	5059.06	0	0.9995	1	21	5017.06
(ΨA)cnpy+smstem, (ΨBA = ΨBa)lgstem+shannon, (pA = rA, pB = rBa = rBA)trail+ugd	5074.45	15.39	0.0005	0.0005	12	5050.45
(ΨA)cnpy+smstem, (ΨBA != ΨBa)lgstem+shannon, (pA = rA, pB = rBa = rBA)trail+ugd	5079.22	20.16	0	0	15	5049.22
(ΨA)cnpy+smstem, (ΨBA != ΨBa)lgstem+shannon, (pA = rA, pB = rBa != rBA)trail+ugd	5083.51	24.45	0	0	18	5047.51
(ΨA)cnpy+smstem, (ΨBA = ΨBa)lgstem+shannon, (pA = rA, pB != rBa = rBA)trail+ugd	5108.39	49.33	0	0	15	5078.39
(ΨA)cnpy+smstem, (ΨBA = ΨBa)lgstem+shannon, (pA = rA, pB != rBa != rBA)trail+ugd	5113.4	54.34	0	0	18	5077.4
(ΨA)cnpy+smstem, (ΨBA != ΨBa)lgstem+shannon, (pA = rA, pB != rBa = rBA)trail+ugd	5116.46	57.4	0	0	18	5080.46
ΨA, ΨBA = ΨBa, pA = rA, pB != rBa = rBA	5130.53	71.47	0	0	5	5120.53
ΨA, ΨBA = ΨBa, pA = rA, pB != rBa != rBA	5131.95	72.89	0	0	7	5117.95
ΨA, ΨBA != ΨBa, pA = rA, pB != rBa = rBA	5132.06	73	0	0	6	5120.06
ΨA, ΨBA != ΨBa, pA = rA, pB != rBa != rBA	5133.63	74.57	0	0	8	5117.63
(ΨA)cnpy+smstem, (ΨBA = ΨBa)lgstem+shannon, (pA = rA, pB = rBa != rBA)trail+ugd	5144.2	85.14	0	0	15	5114.2
ΨA, ΨBA != ΨBa, pA = rA, pB = rBa != rBA	5164.32	105.26	0	0	6	5152.32
ΨA, ΨBA != ΨBa, pA = rA, pB = rBa = rBA	5177.2	118.14	0	0	5	5167.2
ΨA, ΨBA = ΨBa, pA = rA, pB = rBa != rBA	5229.64	170.58	0	0	5	5219.64
ΨA, ΨBA = ΨBa, pA = rA, pB = rBa = rBA	5264.06	205	0	0	4	5256.06

Supplementary Information 5d. Co-occurrence occupancy models describing interactions between rusty-spotted genets (species B) and African golden cats (species A). The best models for all species included the most significant covariates from single-species single-season occupancy models. Covariate abbreviations are explained in Supplementary Information 1. Parameters are explained in the Methods. Models are constructed to test whether parameters of interest equal (=) or not equal (!=) to each other. The best model was selected based on AIC.

**African golden cat – rusty-spotted genet models**	**AIC**	**ΔAIC**	**AIC weight**	**Model Lik.**	**no. param.**	**-2LogLik**
(ΨA)cnpy+smstem, (ΨBA != ΨBa) shannon, (pA = rA, pB != rBa != rBA) trail + ugd	4252.07	0	0.8249	1	19	4214.07
(ΨA)cnpy+smstem, (ΨBA = ΨBa) shannon, (pA = rA, pB != rBa != rBA) trail + ugd	4255.17	3.1	0.1751	0.2122	17	4221.17
(ΨA)cnpy+smstem, (ΨBA != ΨBa) shannon, (pA = rA, pB != rBa = rBA) trail + ugd	4281.42	29.35	0	0	16	4249.42
(ΨA)cnpy+smstem, (ΨBA = ΨBa) shannon, (pA = rA, pB != rBa = rBA) trail + ugd	4289.05	36.98	0	0	14	4261.05
(ΨA)cnpy+smstem, (ΨBA = ΨBa) shannon, (pA = rA, pB = rBa = rBA) trail + ugd	4361.66	109.59	0	0	11	4339.66
(ΨA)cnpy+smstem, (ΨBA != ΨBa) shannon, (pA = rA, pB = rBa = rBA) trail + ugd	4362.24	110.17	0	0	13	4336.24
(ΨA)cnpy+smstem, (ΨBA = ΨBa) shannon, (pA = rA, pB = rBa != rBA) trail + ugd	4365.74	113.67	0	0	14	4337.74
(ΨA)cnpy+smstem, (ΨBA != ΨBa) shannon, (pA = rA, pB = rBa != rBA) trail + ugd	4433.1	181.03	0	0	16	4401.1
ΨA, ΨBA = ΨBa, pA = rA, pB != rBa = rBA	4684.57	432.5	0	0	5	4674.57
ΨA, ΨBA != ΨBa, pA = rA, pB != rBa = rBA	4686.57	434.5	0	0	6	4674.57
ΨA, ΨBA != ΨBa, pA = rA, pB != rBa != rBA	4731.03	478.96	0	0	7	4717.03
ΨA, ΨBA = ΨBa, pA = rA, pB != rBa != rBA	4741.01	488.94	0	0	6	4729.01
ΨA, ΨBA = ΨBa, pA = rA, pB = rBa != rBA	4793.95	541.88	0	0	5	4783.95
ΨA, ΨBA != ΨBa, pA = rA, pB = rBa != rBA	4795.91	543.84	0	0	6	4783.91
ΨA, ΨBA = ΨBa, pA = rA, pB = rBa = rBA	4796.21	544.14	0	0	4	4788.21
ΨA, ΨBA != ΨBa, pA = rA, pB = rBa = rBA	4798.17	546.1	0	0	5	4788.17

Supplementary Information 5e. Co-occurrence occupancy models describing interactions between marsh mongooses (species B) and African golden cats (species A). The best models for all species included the most significant covariates from single-species single-season occupancy models. Covariate abbreviations are explained in Supplementary Information 1. Parameters are explained in the Methods. Models are constructed to test whether parameters of interest equal (=) or not equal (!=) to each other. The best model was selected based on AIC.

**African golden cat – marsh mongoose models**	**AIC**	**ΔAIC**	**AIC weight**	**Model Lik.**	**no. param.**	**-2LogLik**
(ΨA)cnpy+smstem, (ΨBA = ΨBa)smstem, (pA = rA, pB != rBa = rBA)trail+ugd	8806.37	0	0.673	1	14	8778.37
(ΨA)cnpy+smstem, (ΨBA != ΨBa)smstem, (pA = rA, pB != rBa = rBA)trail+ugd	8808.11	1.74	0.282	0.419	16	8776.11
(ΨA)cnpy+smstem, (ΨBA != ΨBa)smstem, (pA = rA, pB != rBa != rBA)trail+ugd	8811.78	5.41	0.045	0.0669	19	8773.78
(ΨA)cnpy+smstem, (ΨBA = ΨBa)smstem, (pA = rA, pB != rBa != rBA)trail+ugd	8857.15	50.78	0	0	17	8823.15
ΨA, ΨBA != ΨBa, pA = rA, pB != rBa = rBA	8952.51	146.14	0	0	6	8940.51
ΨA, ΨBA = ΨBa, pA = rA, pB != rBa = rBA	8962.12	155.75	0	0	5	8952.12
(ΨA)cnpy+smstem, (ΨBA = ΨBa)smstem, (pA = rA, pB = rBa != rBA)trail+ugd	8982.27	175.9	0	0	14	8954.27
(ΨA)cnpy+smstem, (ΨBA != ΨBa)smstem, (pA = rA, pB = rBa != rBA)trail+ugd	8982.96	176.59	0	0	16	8950.96
(ΨA)cnpy+smstem, (ΨBA = ΨBa)smstem, (pA = rA, pB = rBa = rBA)trail+ugd	8983.93	177.56	0	0	11	8961.93
(ΨA)cnpy+smstem, (ΨBA != ΨBa)smstem, (pA = rA, pB = rBa = rBA)trail+ugd	8984.6	178.23	0	0	13	8958.6
ΨA, ΨBA != ΨBa, pA = rA, pB != rBa != rBA	8996.01	189.64	0	0	7	8982.01
ΨA, ΨBA = ΨBa, pA = rA, pB != rBa != rBA	9011.58	205.21	0	0	6	8999.58
ΨA, ΨBA != ΨBa, pA = rA, pB = rBa = rBA	9149.68	343.31	0	0	5	9139.68
ΨA, ΨBA != ΨBa, pA = rA, pB = rBa != rBA	9151.06	344.69	0	0	6	9139.06
ΨA, ΨBA = ΨBa, pA = rA, pB = rBa = rBA	9156.55	350.18	0	0	4	9148.55
ΨA, ΨBA = ΨBa, pA = rA, pB = rBa != rBA	9157.93	351.56	0	0	5	9147.93
